# Rethinking Tertiary Models: Relationships between Growth Parameters of *Bacillus cereus* Strains

**DOI:** 10.3389/fmicb.2017.01890

**Published:** 2017-09-28

**Authors:** József Baranyi, Nathália Buss da Silva, Mariem Ellouze

**Affiliations:** ^1^Institute of Nutrition, University of Debrecen, Debrecen, Hungary; ^2^Department of Physics, Imperial College London, London, United Kingdom; ^3^Department of Chemical and Food Engineering, Federal University of Santa Catarina, Florianópolis, Brazil; ^4^Nestlé Research Center, Lausanne, Switzerland

**Keywords:** cardinal temperatures, Ratkowsky model, Rosso model, reparameterization, *Bacillus cereus*

## Abstract

The maximum specific growth rates of 12 strains, pair-wise belonging to six groups of *Bacillus cereus sensu lato*, were fitted against temperature by a reparametrized version of the model of Ratkowsky et al. (1983). This way, the interpretation of the new parameter set was similar to that of the cardinal-values-model of Rosso and Robinson (2001), both models including the minimum, optimum and maximum temperatures for growth as well as a fourth parameter scaling along the dependent variable. The modularity of the reparametrized version of the Ratkowsky model was utilized to show a so-far undetected relationship between this scaling parameter and the cardinal temperatures, which linked even distant (e.g., mesophilic and psychotropic) strains of *B. cereus*. We propose that the name “tertiary modeling” should be used for investigations like ours, as logically derived from the concepts of “primary” and “secondary” modeling. Such tertiary models may reveal biological relationships between kinetic parameters within a group of strains. It can also be used to create an overarching predictive model for mixed cultures, when different strains grow together but independently of each other.

## Introduction

It is a basic problem in quantitative microbiology whether strains belonging to the same taxonomical group also behave similarly in terms of their kinetics. We investigate this question on *Bacillus cereus sensu lato*, which has a rather complex taxonomy. It is composed of seven closely related phylogenetic groups: *B. cereus sensu stricto* (psychotropic and mesophilic), *B. thuringiensis, B. anthracis, B. weihenstephanensis* (mainly mesophilic), *B. mycoides, B. pseudomycoides* and *B. cytotoxicus*. While strains clustering in group I (*B. pseudomycoides*), group VI (*B. weihenstephanensis*, and *B. mycoides*) and group VII (*B. cytotoxicus*) appear to be different species, the strains of *B. cereus, B. thuringiensis* and *B. anthracis* are spread over groups II, III, IV, and V without formation of clusters (Guinebretiere et al., 2010). On the other hand, comparison of housekeeping genes with MLST (MultiLocus Sequence Typing) and MEE (MultiLocus Enzyme Electrophoresis) suggest that *B. cereus, B. thuringiensis* and *B. anthracis* should belong to a single species (Helgason et al., 2000). Other studies also demonstrated very high sequence similarity of the 16S rRNA gene between them (Sacchi et al., 2002). A generic characterisation of the temperature responses of this complex set of groups would be of special interest since they include psychotropic as well as mesophilic strains.

Temperature is arguably the most influential external environmental factor that affects microbial kinetics. Mathematical modeling for food microbiology also started with describing the effect of temperature on the inactivation of *Clostridium botulinum* in canned food (Bigelow and Esty, 1920; Bigelow, 1921) and most of the early predictive microbiology articles, too, were focused on the thermal inactivation of foodborne bacteria. It was only in the 1980's when the first predictive models describing the effect of temperature on the growth of foodborne micro-organisms were published (Ratkowsky et al., 1983; Rosso et al., 1993). These authors showed that the simplest model describing the effect of temperature on the specific growth rates of various bacteria requires four parameters: the minimum, optimum, maximum temperatures and the optimum specific growth rate. These were called the cardinal values by Rosso et al. (1993). Rosso and Robinson (2001) also used these cardinal values for their model, which was a sort-of “fine-tuning” of the original Rosso model.

Corkrey et al. (2012) assumed a universal thermodynamic constant to explain why the effect of temperature on the specific growth rates of more than 1600 strains of various organisms can be described by an asymmetric delta-shaped function, determined by the above cardinal values. The question naturally rises: is there any relationship between them that could reveal some biological universality? If yes, that would narrow down the parameter space, in which models of bacterial kinetics can move, making their regression to observed data more robust and boosting the confidence in their use for practical predictions. Indeed, Rosso et al. (1993) showed, over several organisms, that the cardinal (minimum, optimum and maximum) temperatures do exhibit strong links over several organisms. This is not surprising, considering that the optimum/maximum temperature is expected to be higher if the minimum temperature is higher. The authors, however, did not find any links between the cardinal temperatures and the optimum specific growth rate. In this paper, we continue this line, investigating whether the latter parameter and the cardinal temperatures can be linked within *B. cereus sensu lato*. For 12 of its strains, belonging to six groups, Carlin et al. (2013) published the maximum specific growth rates, measured at various growth temperatures. We are looking for certain invariants, linking all the six groups, that would explain common patterns in their “growth rate vs. temperature” models.

## Materials and methods

**Figure 1:**
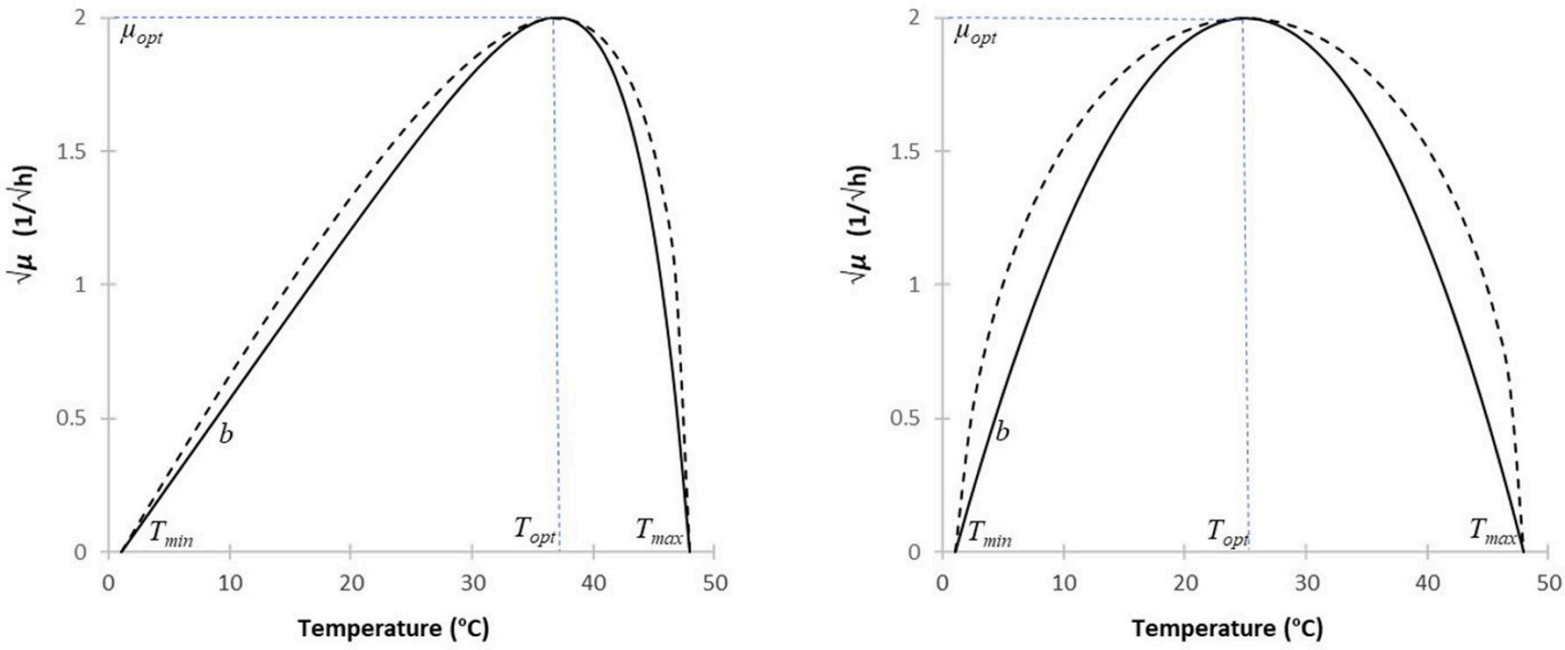
Simulating the Ratkowsky and Rosso models (continuous and broken lines, resp.), to describe the effect of temperature on the square-root of the maximum specific growth rate. The minimum and maximum temperatures were chosen as 1 and 48°C, resp.; the square root of the optimum specific growth rate is 2/h. For most microorganisms, the optimum is much closer to the maximum than to the minimum temperature (first case, *T*_*opt*_ = 37°C). If the optimum is approaching to the mid-point between the minimum and maximum temperatures (second case, *T*_*opt*_ = 25°C), then the slope of the Rosso model, at the extreme temperatures, converges to infinity, making the model less suitable for parameter estimation.

To characterize the growth response of the microorganisms to the temperature, two models have become widely used: that of Ratkowsky et al. (1983) and that of Rosso and Robinson (2001). They both have the shape of an asymmetric delta (Figure 1), with the optimum generally much closer to the maximum than to the minimum growth temperature. In fact, in this case, the two models are hardly distinguishable, though the Ratkowsky model is practically linear for sub-optimal temperatures, while the Rosso model is more noticeably convex from below.

1. The Ratkowsky model is based on the observation that, for a wide range of bacteria, at sub-optimal temperatures, the square root of the maximum specific growth rate, denoted by μ in what follows, linearly depends on the temperature, while it is exponentially decreases to zero as the temperature passes its optimal value:

(1) $$\begin{array}{l} \sqrt{\mu }=b\cdot \left(T-T_{min}\right)\cdot \left(1-e^{c\left(T-T_{max}\right)}\right) \end{array}$$

where *T* quantifies the temperature, *b* and *c* are rate constants, *T*_*min*_, *T*_*max*_ are parameters denoting “nominal” minimum and maximum temperatures for the growth range. We call them nominal because no reliable growth rates can be observed in their neighborhood still they are convenient for interpretation.

2. The cardinal temperature model of Rosso and Robinson (2001), which is a refinement of the model of Rosso et al. (1993), also has four parameters. Generally, its shape is similar to that of the Ratkowsky model but its algebraic form is different. For compatibility, we put it down in a rearranged form so the similarity and difference between the two models can easily be seen:

(2a) $$\begin{array}{l} \sqrt{\mu }=b_{\mu }\cdot \left(T-T_{min}\right)\cdot F\left(T\right) \end{array}$$

where

(2b) $$\begin{array}{l} b_{\mu }=\sqrt{\frac{\mu_{opt}}{\left(T_{opt}-T_{min}\right)}} \end{array}$$

(2c) $$\begin{array}{l} F\left(T\right)=\sqrt{\frac{\left(T_{max}-T\right)}{\left(T_{opt}-T_{min}\right)\left(T_{opt}-T\right)+\left(T_{max}-T_{opt}\right)\left(T-T_{min}+T-T_{opt}\right)}} \end{array}$$

Here, the minimum and maximum growth temperatures are as above, while μ_*opt*_ and *T*_*opt*_ are the location and value of the function at optimum temperature. Understandably, the easy microbiological interpretation of its parameters has contributed to the popularity of the model.

Both models are interpreted for *T*_*min*_ < *T* < *T*_*max*_ temperatures only. Besides, it is also evident from their structures that both the *b* and *b*_μ_ parameters control the respective slopes around the minimum temperature, thus playing the role of scaling along the vertical axis. The remaining terms only depend on temperature differences. The first part of both models is linear, which is modified by a factor that, as the temperature increases toward its maximum, exponentially converges to zero in Equation (1), while its equivalent, F(*T*), shows a square-root-like convergence to zero in Equation (2a). The difference between the two models becomes obvious if the optimum temperature is close to the mid-point between the minimum and maximum temperature. In that case, the denominator in the last factor of the Rosso model is becoming closer and closer to zero, which produces an increasingly pronounced convex shape at low temperatures (Figure 1). For the exact *T*_*opt*_ = (*T*_*min*_+*T*_*max*_)/2 case, the Rosso model is not interpretable, the denominator in Equation (2a) becoming zero.

For most micro-organisms, the optimum is much closer to the maximum temperature, in which case the two models are very close to each other (Figure 1). However, when using them for regression, there can be significant differences between them in terms of robustness (i.e., sensitivity to noisy data) and uncertainty of the parameter estimates. Such investigations have been carried out by Ratkowsky (2003), though using the original model of Rosso et al. (1993). It is out of the scope of this paper to repeat his analysis using the model of Rosso and Robinson (2001); let it suffice to note that (as a principle in numerical analysis), estimating parameters in the denominator is less advantageous than estimating those in the numerator. This is one of the reasons why we chose the Ratkowsky model for our purposes. The other reason is the modularity of Equation (1). Its significance is that, if we have a robust, well-working model for sub-optimal temperatures [i.e., without the last factor in Equation (1) causing the non-linearity], then new super-optimal temperature data can just be added to the regression so that the already calculated *b* and *T*_*min*_ parameters can remain fixed. That is, we can easily ensure that the location and value of the optimum growth rate have no effect on the former estimates of the *b* and *T*_*min*_ parameters. This is not the case with the Rosso model, as shown by the formula for *b*_μ_ in Equation (2b).

Further increasing the comparability of the two models, we can make sure, by some numerical techniques (see below and the Implicit function problem in the Supplementary Material), that the parameter interpretation of the Ratkowsky is equivalent to that of the Rosso model. Take the derivative, according to the temperature, of the right-hand side of the Ratkowsky model. It should be zero for the *T*_*opt*_ optimum temperature, from which:

(3) $$\begin{array}{lll} c\left(T_{max}-T_{opt}\right) & = & \ln\left[1+c\left(T_{opt}-T_{min}\right)\right],\\ \text{ where }c\;>0\text{ and }T_{min}<T_{opt}<T_{max} \end{array}$$

From this formula, an *R* function and its inverse can be constructed by a numerical algorithm, so that

(4) $$\begin{array}{l} c=R\left(T_{opt};T_{min},T_{max}\right)\;and\;T_{opt}=R^{-1}\left(c;T_{min},T_{max}\right) \end{array}$$

Apart from the *c* = 0 trivial solution, these functions are uniquely defined under the condition that *T*_*opt*_–*T*_*min*_ > *T*_*max*_–*T*_*opt*_, i.e., the sub-optimal temperature range is greater than the super-optimal.

In the Supplementary Material, we also provide a short and robust algorithm in Visual Basic to calculate the *R* and *R*^−1^ functions. Thus, the Ratkowsky model can be written in the form,

(5) $$\begin{array}{l} \sqrt{\mu }=b\left(T-T_{min}\right)\cdot \left(1-e^{R\left(T_{opt},\;T_{min},\;T_{max}\right)\cdot \;\left(T-T_{max}\right)}\right) \end{array}$$

In this arrangement of the Ratkowsky model, the reparametrized coefficient *c* = *R(T*_*opt*_*, T*_*min*_*, T*_*max*_) contains the cardinal temperature values, just as the F(*T*) factor in Rosso's model (Equation 2a). As mentioned, the role of the coefficient *b* is analogous to that of *b*_μ_ in the Rosso model; both are just scaling the remaining, temperature-dependent part of the respective equations. Therefore, the formulation of the Ratkowsky model by Equation (5) is as much analogous as possible with that of the Rosso model, the computational benefits of which was utilized when creating Figure 1.

Carlin et al. (2013) studied two different strains from each of the six groups of *B. cereus*. We analyzed the datasets, available for each strain in the Supplementary Material, in two steps. First, we fitted the √μ values for a suboptimal temperature region (*T* ≤ 30°C) by the first, linear module of Equation (5), what we will call the “truncated” Ratkowsky model. The result will be called the “Ratkowsky-lines.” The reason for carrying out this first step, only using the truncated Ratkowsky model, is that the super-optimal temperature data are much more erratic and fitting them simultaneously can corrupt the estimates of the *T*_*min*_, *b* parameters, which can be accurately estimated even by just using the much less noisy sub-optimal data. In the second step, we fixed the *T*_*min*_ and *b* parameters obtained in the first step and fitted only the remaining *T*_*opt*_ and *T*_*max*_ parameters of Equation (5). Then we studied whether links can be detected between the obtained strain-specific parameter estimates. The importance of such links is not only that they might reflect genome-level similarities but that they can also be used, for example, to develop overarching models for mixed cultures.

## Results

**Figure 2:**
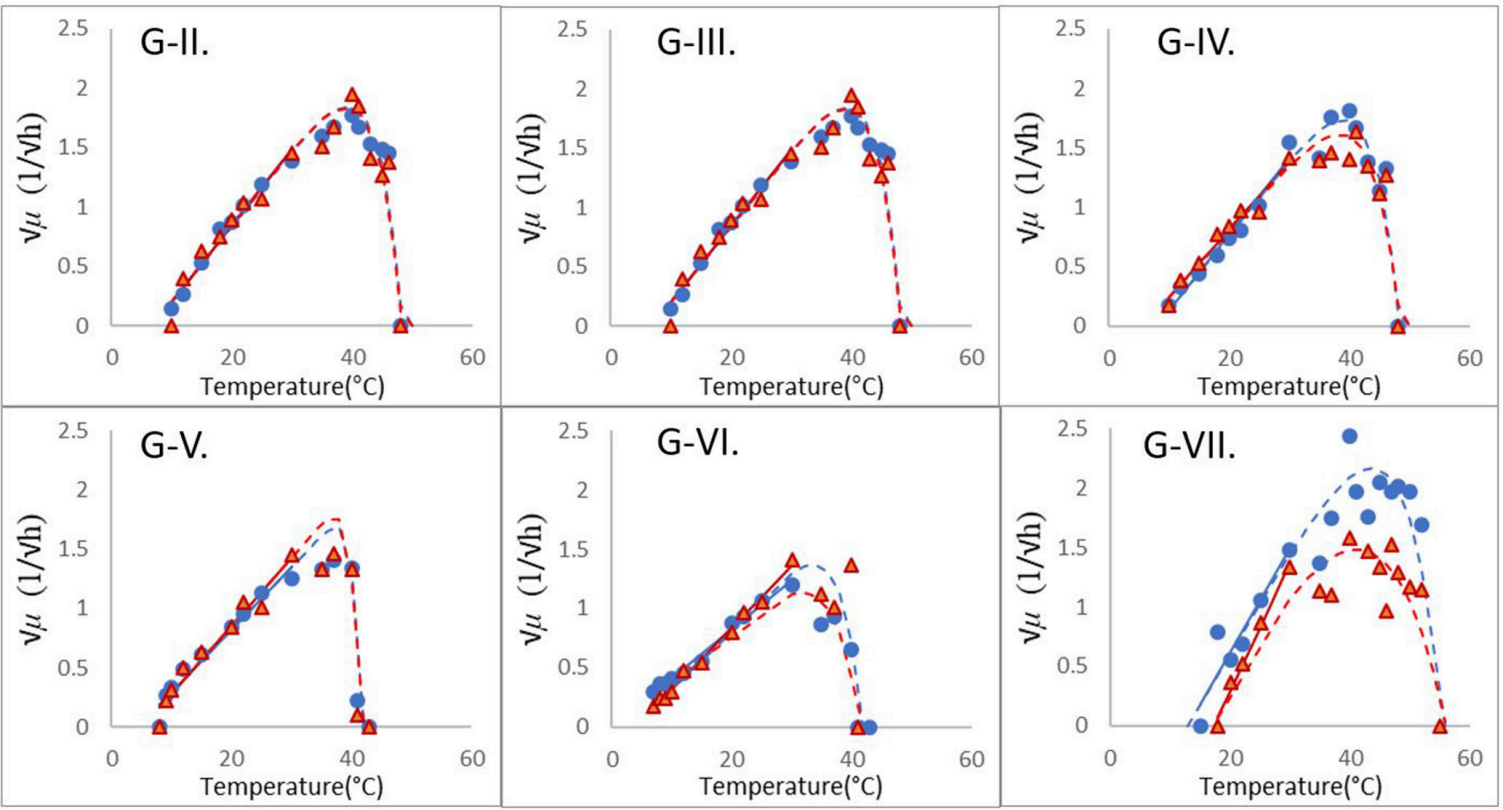
Modular fitting of the Ratkowsky model to the √μ *vs*. temperature data of six groups of *B. cerus*; see Carlin et al. (2013), as in Table 1. In the first step, the linear module of Equation (1) was fitted to the less erratic, sub-optimal data (≤ 30°C). The obtained *T*_*min*_ and *b* parameters of the resultant linear model (continuous straight lines) were then fixed and only the *T*_*opt*_, *T*_*max*_ parameters were determined in the second step.

**Figure 3:**
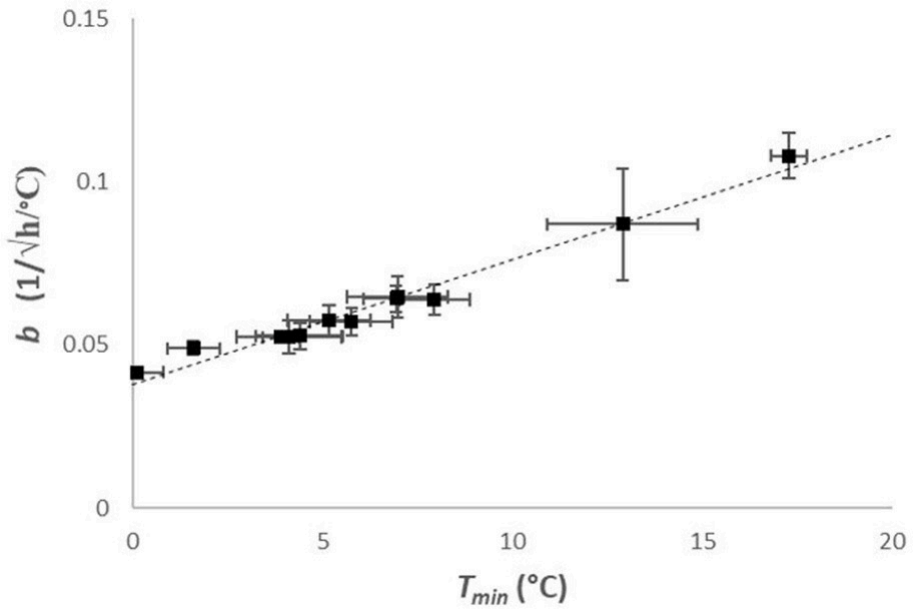
*B-line*: The estimated *b* and *T*_*min*_ values (represented with their standard errors here) are in a strong linear relationship across the six groups. The *b*= β·(*T*-*T*_*0*_) equation (*R*^2^ = 0.97), with β = 0.0038 ± 0.0002 (1/√h/°C^2^) and *T*_0_ = −9.9 ± 0.5 (°C), holds from psychotropic to mesophilic strains.

Table 1 shows the estimates and their standard errors obtained from the procedure above. Figure 2 shows the square-root values of the maximum specific growth rates published in the Supplementary Information of Carlin et al. (2013), as well as the fitted Ratkowsky lines and the full models for each of the 12 strains, divided into the six groups of *B. cereus*. The *b*-slopes of the Ratkowsky-lines show a surprisingly strong linear dependence on the *T*_*min*_ temperatures (Figure 3). In fact, the

(6) $$\begin{array}{l} b=\beta \left(T_{min}-T_{\text{0}}\right) \end{array}$$

linear model (*R*^2^ = 0.97), that we will call *B-line* in what follows, is supported by data from psychotropic as well as mesophilic strains.

**Table 1:** Fitting the reparametrized Ratkowsky-model to the data generated for 12 strains of six groups of *B. cereus*, published in the Supplementary Information of Carlin et al. (2013).

		***T_min_*** and std. err. (°C)		**b** and std. err. (1/√h)/°C		***T*_*opt*_** and std. err. (°C)		**T_*max*_** and std. err. (°C)	
**G.–II**.	RIVM-BC120	**1.59**	0.696	**0.0489**	0.00211	**37.79**	0.517	**41.08**	0.083
	NVH-0860-01	**4.11**	1.371	**0.0524**	0.00516	**34.83**	0.576	**40.96**	0.142
**G.–III**.	F4810/72	**6.94**	0.854	**0.0642**	0.00404	**39.41**	0.420	**48.28**	0.252
	F837/76	**6.97**	1.334	**0.0647**	0.00636	**39.13**	0.448	**48.20**	0.266
**G.–IV**.	F4430/73	**7.95**	0.943	**0.0639**	0.00474	**39.09**	0.414	**48.20**	0.255
	ATCC-14579	**5.76**	1.085	**0.0571**	0.00423	**38.38**	0.470	**48.40**	0.343
**G.–V**.	F2769/77	**4.40**	1.147	**0.0527**	0.00420	**37.60**	0.546	**41.13**	0.104
	NVH-141/1-01	**5.17**	1.080	**0.0576**	0.00452	**37.15**	0.644	**41.08**	0.120
**G.–VI**.	KBA-B4	**0.10**	0.714	**0.0416**	0.00171	**31.64**	0.590	**41.43**	0.310
	I-21	**3.90**	0.463	**0.0523**	0.00172	**33.55**	1.128	**41.83**	0.709
**G.–VII**.	NVH-391/98	**12.9**	1.969	**0.0870**	0.01710	**43.59**	0.484	**55.48**	0.538
	NVH-883/00	**17.28**	0.468	**0.1080**	0.00707	**41.11**	0.416	**56.14**	0.955

It is vital to see that Equation (6) describes a *biological* relationship and not a regression-related correlation between the *b* and *T*_*min*_ parameters. The latter one would indicate that the numerical estimation procedure for one parameter influences the estimation of the other one. This could be a result of over-parametrisation and could be demonstrated by simulating noisy measurements around the model for a single strain. However, what we observe here could not be detected based on one or two strains. Our finding is a relationship between the parameters detected when considering several strains of *B. cereus sensu lato*. In other words, the four kinetic parameters of a strain do not scatter arbitrarily in the 4D-space. This is evident for the cardinal temperatures, but has so far been unknown for the *b* parameter (hence for the optimum growth rate, too).

**Figure 4:**
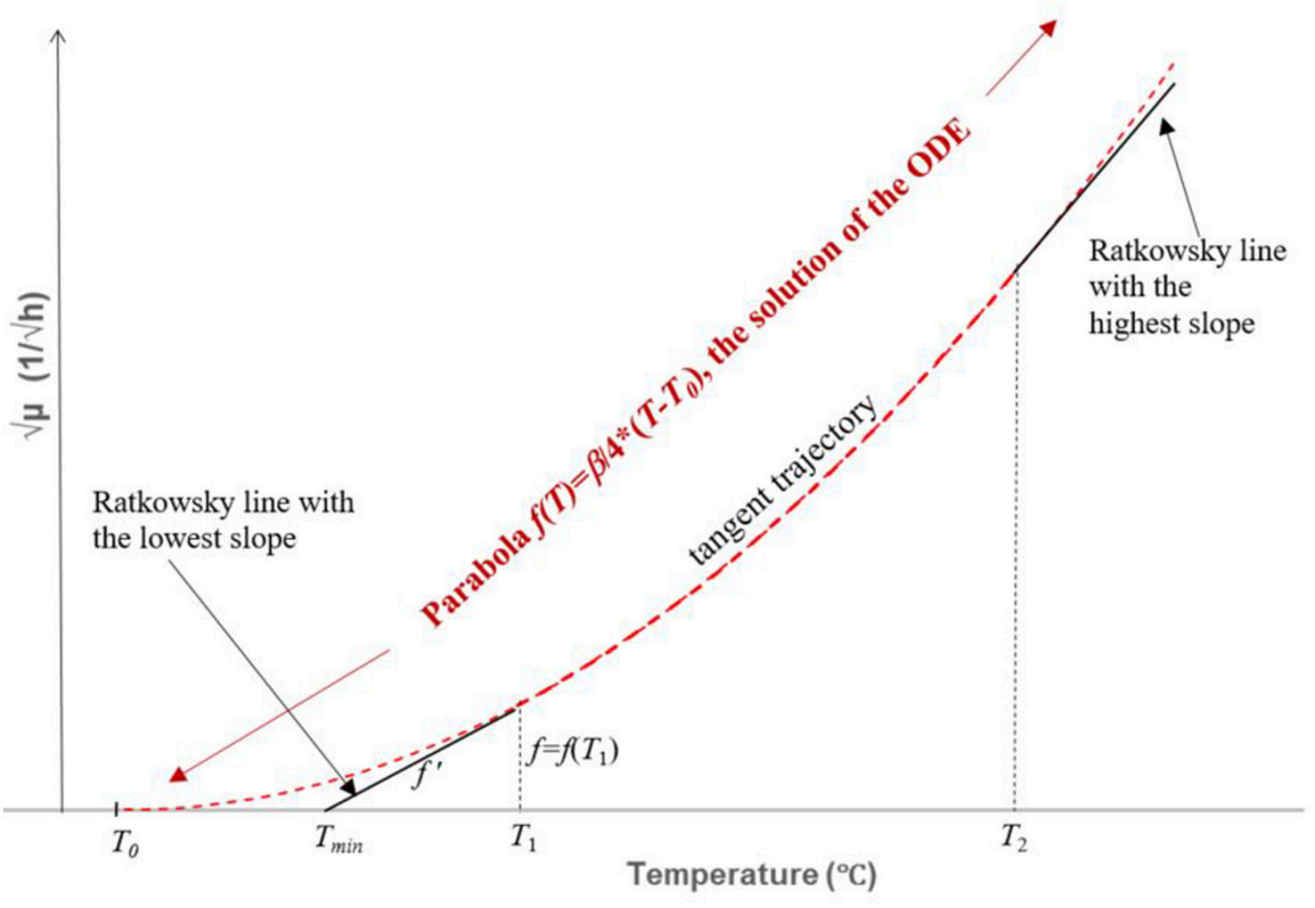
Construction and solution of the ordinary differential equation (ODE) described by Equation 7. It is part of the envelope representing the square root of the maximum specific growth rate of the fastest strain as a function of the temperature. The ODE describes a curve in the (*T*_1_ *T*_2_), interval to which each Ratkowsky-line is a tangent. The relationship between the *f* (*T*) value and the *f*'(*T*) slope (*T* = *T*_1_ in the figure) can be written up by means of the obtained linear relationship (the *B-line*) between the two parameters of the linear (truncated) Ratkowsky model. The solution is the parabola *b* = (β/4)·(*T*-*T*_0_)^2^, represented by a broken red curve inside and outside the (*T*_1_, *T*_2_) interval. The *T*_1_, *T*_2_ values are those where two tangents (black continuous straight lines), one with the lowest and one with the highest slope, respectively, touch the parabola. These two tangents also serve as smooth continuations of the parabola for *T* < *T*_1_ and *T* > *T*_2_ values, thus becoming part of the wanted envelope (see Figure 5).

Notice that, by means of the *B-line*, a tangent-trajectory for the Ratkowsky-lines can be calculated as follows:

At each *T* = *T*_1_ point of the *f* = *f* (*T*) trajectory, the tangent has the value of *f*', which is, according to the *B-line*, a linear function of the intercept of the tangent with the horizontal axis (Figure 4). Therefore,

(7) $$\begin{array}{l} f^{\prime }=\beta \cdot \left(T_{min}–T_{\text{0}}\right)=\beta \cdot \left(T-f/f^{\prime }–T_{\text{0}}\right) \end{array}$$

**Figure 5:**
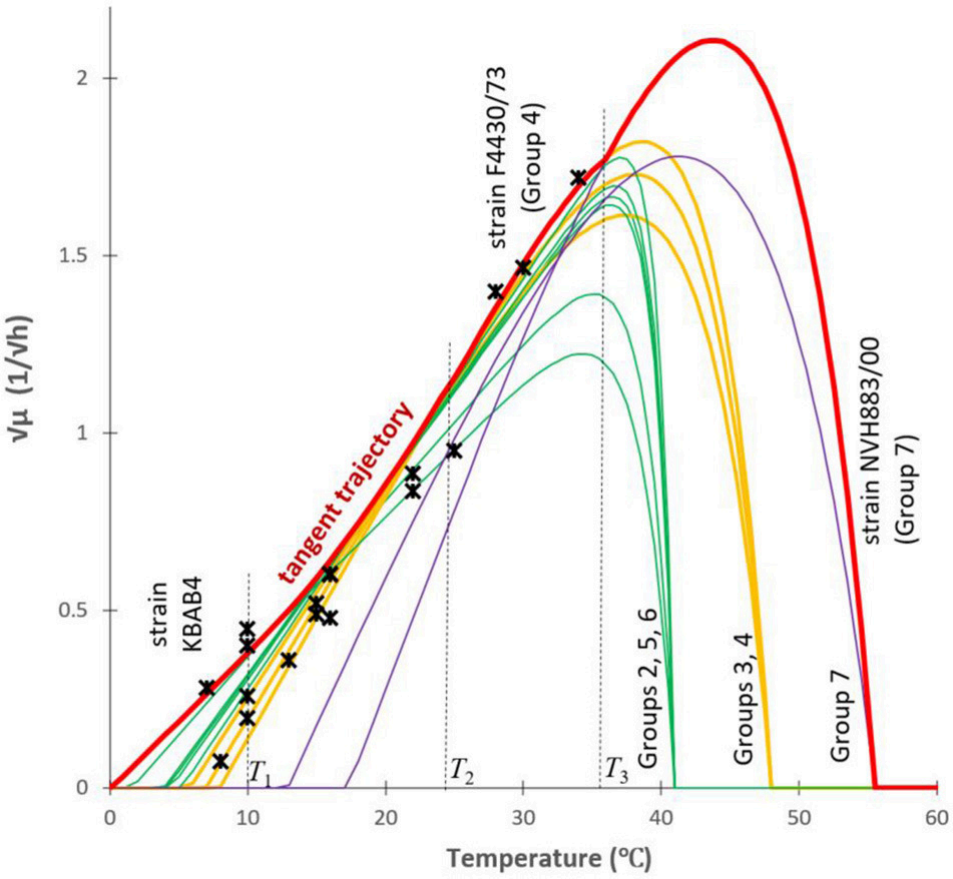
An over-arching envelope (in red) for the “square root of the maximum specific growth rate *vs*. the temperature” models for all the studied strains. The relationship between the *T*_*min*_ and *b*-values were used to create a tangent trajectory (a parabola, see Figure 4) for the Ratkowsky-lines of all the studied strains in the (10°C = *T*_1_ < *T* < *T*_2_ = 24°C) region of temperature. Outside this interval, the Ratkowsky lines for Group 6 (*T* < *T*_1_ = 10°C), for Group 4 (24°C = *T*_2_ < *T* < *T*_3_ = 36°C) and the model for Group 7 (*T* > 36°C) form a generic model (thick red curve) for the dominant strain for any temperature point. Stars are independent data from ComBase.

This is an ordinary differential equation (ODE) for the *f(T)* tangent trajectory, where the dependence of *f'(T)* on *f(T)* is given in an implicit form. It could be expressed for *f*' but the structure of the obtained ODE would be rather complicated to solve in an algebraic way. However, remember that the parabola is known to have such proportionality properties therefore it is worth testing a parabolic solution. Indeed, as can be checked by substitution, the parabola,

(8) $$\begin{array}{l} f\left(T\right)=\frac{\beta }{4}\cdot {\left(T-T_{0}\right)}^{2} \end{array}$$

satisfies the ODE with the *f(T*_0_*)* = 0 initial value (which requirement can be readily seen). This is the equation for the parabola shown in Figure 4. It can be used to create a generic model that represents the maximum specific growth rate of the dominant strain of a mixed culture, useful to produce a *safe* prediction when the identity of the *B. cereus* strains is not known (Figure 5). At low temperatures (*T* < *T*_1_ = 10°C), this strain is from Group 6; at higher but still suboptimal temperatures (24°C = *T*_2_ < *T* < *T*_3_ = 37°C), it is from Group 4. For 10°C = *T*_1_ < *T* < *T*_2_ = 24°C, the above parabola describes a tangent trajectory in the region, where practically any strains from Group 2–6 can be dominant (Figures 4, 5). Above *T*_3_ = 37°C, the Group 7 takes over the role of the dominant strain.

The from-below-concave (and not linear) trend observed at sub-optimal temperatures can are compared to data from the ComBase database (www.combase.cc–Figure 5). There, the records with keys starting B284 and B224 (sources: FSA_FMBRA and FSA_IFR) contain growth curves of *B. cereus* strains measured by plate count methods as described by Sutherland et al. (1996). We selected those records where the specific growth rates were generated in culture medium, at optimal pH (6.8 < *pH* < 7.2) and water activity (added *NaCl* < 1.5%). As described in the respective “details” field in the database, apart from the temperature, the measurements of mixed cultures of *B. cereus* were carried out in optimal conditions. The square root values of the specific growth rates were plotted on Figure 5, which validated our predicted trajectory. The from-below-concave pattern is the result of the fact that the identity of the dominant organism in a mixed culture depends on the temperature. This explains why sometimes the expected linear trend of Ratkowsky is not observable with mixed cultures, when plotting the square root of measured maximum specific growth rates against temperature.

Figure 5 also demonstrates how the cardinal temperatures of the studied *B. cereus* models can be grouped. The optimum and maximum temperatures of Groups 2, 5, and 6 form one cluster, the Groups 3 and 4 do another one. Group 7 grows at high temperatures and it is further away from the other two clusters. In some way, it is surprising that the minimum temperatures did not follow this clustering, except for Group 7, for which both the minimum and maximum temperatures were high.

## Discussion

Here first we showed analogies as well as differences between the Ratkowsky and Rosso models. Equation (5) showed that the Ratkowsky model can also be arranged to have the cardinal values. We pointed out that, for the common case, when the optimum is much closer to the maximum than to the minimum temperature, both models perform well, in terms of goodness of fit, but the Ratkowsky model also has an embedded, linear sub-model, that is useful for regressing data at lower temperatures. Note that Buss da Silva et al. (2017) used this sub-model, too, to describe the effect of sub-optimal temperatures on the maximum specific growth rates of *B. cereus* grown in Reconstituted Infant Formulae, but with the logarithm (instead of the square root) link function for the growth rate. The modularity of the Ratkowsky model was utilized to point out a relationship between the *b* and *T*_*min*_ parameters of 12 strains belonging to *B. cereus sensu lato*.

Our study raises a need to clarify the use of the term “tertiary model” in predictive microbiology. The focus of the discipline is how a certain measure (cell and/or metabolite concentration, etc.) of an organism can be predicted, for a given nutrient source (laboratory medium or food). Primary models describe the time-variation of the response assuming everything else (organism, nutrient source, temperature, etc.) is fixed. Secondary models describe the effect of the environmental variables, such as temperature, on the primary model parameters, assuming the nutrient source and the organism are fixed. Following this logic, the name “tertiary model” should be used for patterns in the parameters of the secondary models as a function of the organism and the nutrient source. Unfortunately, the term was introduced differently (Buchanan, 1993), for the composite of the previous two kinds of models, when implementing them for computer applications. Hereby we propose to use it in the logical way described above. For example, Buss da Silva et al. (2017) carried out systematic experiments showing that there is a constant ratio (the so-called bias factor) between the maximum specific growth rates in culture medium and in a specific food (Reconstituted Infant Formulae), if all other conditions are the same. The constant bias factor is a simple “tertiary” model meant in the above sense, inasmuch it describes a relationship between culture-medium-based and food-based secondary models.

Our study is also a tertiary modeling step, in this sense: how do the secondary model parameters depend on the strain belonging to a defined set of organisms? At the first instance the question looks odd, since the identity of the strain is a category (not continuous) variable. Namely, going back to the original ideas, the primary and secondary modeling are based on an important physical property of the considered variables: that their variation is quantified by continuous metrics, therefore if two values (like two timepoints) or are close to each other, then the values of the modeled variable are also expected to be close to each other. This was true for the bias factor as “tertiary modeling,” too: if the medium changes a little than the response (a secondary model parameter) should change a little only.

For our case, the taxonomical links between the strains can define the needed “closeness” concept and it does make sense to ask how the secondary model parameters depend on the strains, for which taxonomy metrics have been defined. Our “B-line” is a simple “tertiary” model, according to the definition above (in which sense also we will use the term). For example, Baranyi et al. (1996) also reported on a tertiary model, a strong link between the secondary model coefficients of four different members of a mold, the genus *Aspergillus*. There, the authors showed that the effect of water activity on the radial growth rate was well described by a triplet of parameters for the studied four species (*A. flavus, A. oryzae, A. parasiticus*, and *A. nomius*); however, these triplets were not scattered in their 3D space but lied along a straight line there, reducing their total degree of freedom by 2. The coefficients of this straight line in the 3D space are, according to our use of the term, tertiary model parameters. The comprehensive study of Corkrey et al. (2012), was also tertiary modeling, where the authors claim that a universal thermodynamic constant can explain why many micro-organisms has secondary models of the same asymmetric delta shape. Such tertiary models can be used not only to create over-arching predictive models for mixed cultures, but also to predict kinetic properties of possible new strains to be detected in the future.

Whether our tertiary model for *B. cereus* has a deeper, genome-level bases, and what other organisms may be suitable for such study, are open questions. It is not easy to find appropriate data, because it would need a systematically designed series of experiments using strains of the same species whose cardinal temperatures are relatively far from each other. Since we are not aware of such publicly available datasets, it remains to be seen whether our findings are specific to *B. cereus* only, or characteristic to other species, too.

## Author contributions

JB: Conception, Execution, Writing up. NB: Conception, Writing up. ME: Writing up.

### Conflict of interest statement

The authors declare that the research was conducted in the absence of any commercial or financial relationships that could be construed as a potential conflict of interest.
