# Supplementary material for: Rethinking Tertiary Models: Relationships between Growth Parameters of Bacillus cereus Strains
Source: Front Microbiol. 2017 Sep 28;8:1890. doi: 10.3389/fmicb.2017.01890 (PMC5627028; doi:10.3389/fmicb.2017.01890)
Supplement: Supplementary file 1 [file DataSheet1.docx]

**Supplementary Information**

1. **Statement**: Solution of the ODE

| $f^{'}=\beta*(T-\frac{f}{f^{'}}-T_{0})$  for *f=f(T)* is  $f=\frac{\beta}{4}*\left( T-T_{0} \right)^{2}$ | (1) |
| --- | --- |
| **Proof**: Considering the above formula for *f* , calculate its derivative: *f’=(β/2)(T-T_0_)*. Substitute *f* and *f’* in the ODE. Note that *T-T_0_* > 0 : |  |
| $\frac{\beta}{2}*\left( T-T_{0} \right)=\beta*\left[ T-\frac{\frac{\beta}{4}*\left( T-T_{0} \right)^{2}}{\frac{\beta}{2}*\left( T-T_{0} \right)}-T_{0} \right]$ | (2) |
| $\frac{\beta}{2}*\left( T-T_{0} \right)=\beta*\left( T-\frac{{T-T}_{0}}{2}-T_{0} \right)$ | (3) |
| $\frac{\beta}{2}*\left( T-T_{0} \right)=\frac{\beta}{2}*\left( T-T_{0} \right)$ | (4) |

**Implicit function problems:**

2a. Express *T_opt_*  by means of the positive *c* from the equation

*c*(*T_max_ - T_opt_*) = ln [ 1+ *c*(*T_opt_ – T_min_*) ]

where *T_min_ , T_max_* are constant in such a way that

*T_min_ < T_opt_ < T_max_*

2.b. As above but express *c* by means of *T_opt_* from the same equation, noting that not only

*T_min_ < T_opt_ < T_max_*

but also

*T_max_ -T_opt_ < T_opt_ -T_min_*

**Numerical solutions**

2.a. On the (*T_opt_ , y*) plane, the *y* = *c*(*T_max_ - T_opt_*) function decreases linearly from (0, *cT_max_*) to

(*T_max_*, 0). It has to cross the *y* = ln [ 1+ *c*(*T_opt_ – T_min_*) ] curve within the (0,*T_max_*) interval, because the latter curve is increasing in a strictly monotonous manner from (0,0)

2.b. On the (*c , y*) plane, the *y* = *c*(*T_max_ - T_opt_*) function increases linearly from (0,0), at the rate (*T_max_ - T_opt_*) . The *y* = ln [ 1+ *c*(*T_opt_ – T_min_*) ] also increases from the origin, but along a trajectory that is convex from below. The derivative of the latter one is *T_opt_ – T_min_* at the origin, which is bigger than the (constant) slope of the other (convex), function, therefore they must cross each other at another point, too, apart from the origin when *c*=0.

An R function and its inverse is shown below implementing the numerical method of bisection, so that

*c* = *Rconts*(*T_opt_; T_min,_*, *T_max_*) and *T_opt_* = *Rinv*(*c; T_min,_*, *T_max_*)

Function Rconst(Topt, Tmin, Tmax)

If Not (Tmin < Topt And Topt < Tmax And Topt - Tmin > Tmax - Topt) Then Stop

eps = 0.000001: xLow = 1: xHi = xLow * 2

check:

If signx(fx(xLow, Topt, Tmin, Tmax)) = signx(fx(xHi, Topt, Tmin, Tmax)) Then

xLow = xLow / 2: xHi = 2 * xHi: If xHi > 100 Then Stop

GoTo check

End If

loopBeg:

xMid = (xLow + xHi) / 2

fxMid = fx(xMid, Topt, Tmin, Tmax)

If Abs(fxMid) < eps Then GoTo LoopEnd

If signx(fx(xLow, Topt, Tmin, Tmax)) = signx(fxMid) Then xLow = xMid

If signx(fxMid) = signx(fx(xHi, Topt, Tmin, Tmax)) Then xHi = xMid

GoTo loopBeg

LoopEnd:

Rconst = xMid

End Function

Function Rinv(c, Tmin, Tmax)

If Not (Tmin < Tmax And c > 0) Then Stop

eps = 0.000001: xLow = (Tmin + Tmax) / 2: xHi = Tmax

loopBeg:

xMid = (xLow + xHi) / 2

fxMid = fx(c, xMid, Tmin, Tmax)

If Abs(fxMid) < eps Then GoTo LoopEnd

If signx(fx(c, xLow, Tmin, Tmax)) = signx(fxMid) Then xLow = xMid

If signx(fxMid) = signx(fx(c, xHi, Tmin, Tmax)) Then xHi = xMid

GoTo loopBeg

LoopEnd:

Rinv = xMid

End Function

Function fx(c, Topt, Tmin, Tmax)

fx = c * (Tmax - Topt) - Log(1 + c * (Topt - Tmin))

End Function

Function signx(x)

If x > 0 Then signx = 1

If x = 0 Then signx = 0

If x < 0 Then signx = -1

End
